# Correction: Multi-pronged molecular insights into flavonoid-mediated inhibition of squalene epoxidase: a pathway to novel therapeutics

**DOI:** 10.1039/d5ra90096d

**Published:** 2025-08-28

**Authors:** Emadeldin M. Kamel, Sarah I. Othman, Hassan A. Rudayni, Ahmed A. Allam, Al Mokhtar Lamsabhi

**Affiliations:** a Chemistry Department, Faculty of Science, Beni-Suef University Beni-Suef 62514 Egypt emad.abdelhameed@science.bsu.edu.eg; b Department of Biology, College of Science, Princess Nourah Bint Abdulrahman University P. O. Box 84428 Riyadh 11671 Saudi Arabia; c Department of Biology, College of Science, Imam Mohammad Ibn Saud Islamic University Riyadh 11623 Saudi Arabia; d Department of Zoology, Faculty of Science, Beni-Suef University Beni-suef 65211 Egypt; e Departamento de Química Módulo 13, Universidad Autónoma de Madrid, Campus de Excelencia UAM-CSIC Cantoblanco 28049 Madrid Spain; f Institute for Advanced Research in Chemical Sciences (IAdChem), Universidad Autónoma de Madrid 28049 Madrid Spain

## Abstract

Correction for ‘Multi-pronged molecular insights into flavonoid-mediated inhibition of squalene epoxidase: a pathway to novel therapeutics’ by Emadeldin M. Kamel *et al.*, *RSC Adv.*, 2025, **15**, 3829–3848, https://doi.org/10.1039/D4RA09076D.

The authors regret that their related work, cited here as ref. 1, was not cited in the original article. Ref. 1 was undergoing peer review at the same time as this article. Although both projects interrogate squalene epoxidase (SQLE) inhibition, each employs a unique, non-overlapping compound library designed around separate SAR hypotheses that emerged from our initial virtual-screening campaign. Ref. 1 focuses on alkaloids, whereas this article focuses on flavonoids.

The Royal Society of Chemistry apologises for these errors and any consequent inconvenience to authors and readers.
